# Evaluation on Anti-hepatitis Viral Activity of *Vitis vinifer* L

**DOI:** 10.3390/molecules15107415

**Published:** 2010-10-22

**Authors:** Tao Liu, Jun Zhao, Haibo Li, Long Ma

**Affiliations:** 1 College of Public Health, Xinjiang Medical University, Urumqi, 830011, China; 2 Xinjiang Key Laboratory for Research and Development of Uighur Medicine, Institute of Materia Medica of Xinjiang, Urumqi, 830002, China; E-Mail: zhaojun21cn@163.com (J.Z.); 3 Nantong Center for Disease Control and Prevention, Nantong, 226007, China; E-Mail: lihaibo1972@163.com (H.L.)

**Keywords:** *Vitis vinifer* L, anti-hepatitis viral activity, orthogonal design

## Abstract

Suosuo grape (*Vitis vinifer* L) is traditionally used as a therapeutic agent for measles and hepatitis by the ethnic Uighurs. This work aimed to investigate the anti-HBV effect of total triterpene (VTT), total flavonoids (VTF) and total polysaccharides (VTP) from Suosuo grape, and their synergistic effects were also tested. The viral antigens of cellular secretion, HBsAg and HBeAg, were determined by enzyme linked immunosorbent assay (ELISA).The quantity of HBV-DNA released in the supernatant was assayed by real-time PCR. It was found that it effectively suppressed the secretion of HBsAg and HBeAg from HepG2.2.15 cells in a dose-dependent manner, as well as the HBV DNA. The results of orthogonal design experiment showed that the combination of VTT 20 μg/mL, VTF 50 μg/mL and VTP 50 μg/mL had the best optimistic inhibitory effects on HBeAg secretion.

## 1. Introduction

Hepatitis B virus (HBV) infections remain a major public health problem for nearly 350 million people. HBV infections frequently results in acute and chronic hepatitis and may develop into liver cirrhosis and hepatocellular carcinoma [1]. Although immunization against HBV has been widely used in preventing viral infections, current therapies for these diseases have limited efficacy, therefore there is still an urgent need for the development of effective antiviral agents to eliminate HBV or to inhibit HBV proliferation in carriers [2]. In recent years, people have paid close attention to active anti-viral compounds from traditional medicine or natural products such as oleanolic acid, silymarin, and schizandrin [3,4] because of their better effects.

Suosuo grape (*Vitis vinifera* L) is widely grown in Xinjiang Province in China, and its fruit has been used as a folk medicine for hepatitis and pedo-measles [5]. Previous studies showed that total triterpene (VTT), total flavonoids (VTF) and total polysaccharides (VTP) from Suosuo grape had significantly protective effects against immunological liver damage induced by Bacille Calmette- Guerin (BCG) plus lipopolysaccharide (LPS) *in vitro* and *in vivo*, and its mechanism might be related to clearance of free radicals, decrease of lipid peroxidation products, protection of hepatocytes membranes, improvement of hepatocyte function and regulation of the secretion of immune cytokines and expressions of apoptosis genes [6,7,8,9]. The aim of this work was to investigate the synergistic inhibitory effects of VTT, VTP and VTF from Suosuo grape on HBV *in vitro*. 

## 2. Results and Discussion

### 2.1. Toxic effect of extracts from suosuo grape on Hep G2.2.15 cells

The cytoxicity test results showed that the TC_50_ of VTT, VTP, VTF and 3-TC were 160.61, 1412.85, 284.91 and 244.97 μg/mL, respectively.

### 2.2. Inhibitory effect of extracts from suosuo grape on the expression of HBsAg, HBeAg and HBV DNA replication

**Figure 1:**
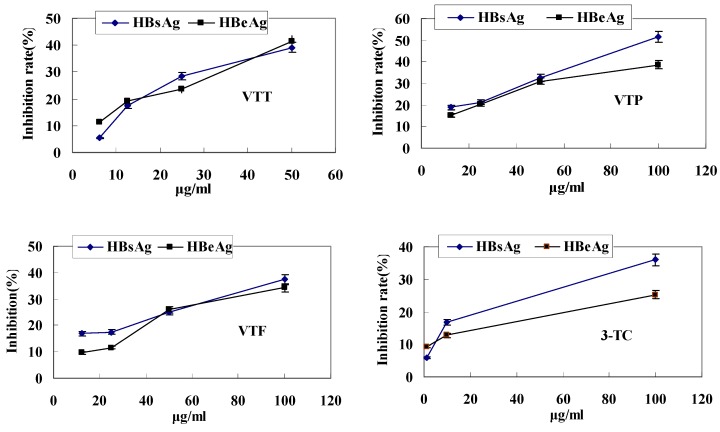
Inhibitory effect of VTP, VTT, VTF and 3-TC on HBsAg and HBeAg secretion.

In this work, the inhibitory effect of extracts from suosuo grape on the expression of HBsAg, HBeAg and HBV DNA replication were investigated, and the results are illustrated in Figure 1. The VTT, VTF and VTP from suosuo grape were able to inhibit the expression of HBsAg, HBeAg, and HBV DNA replication *in vitro* in a dose-dependent manner. The median effective dose (IC_50_) of inhibition HBsAg were 62.54, 112.69 and 327.56 µg/mL, the individual therapeutic indexes of HBsAg were 2.57, 12.54 and 0.87. The median effective doses (IC_50_) of HBeAg inhibition were 89.28, 204.46 and 215.34 µg/mL, the individual therapeutic indexes of HBeAg were 1.80, 6.91 and 1.32 (Table 1). The inhibitory effect on HBV DNA was enhanced with the increasing concentration of extracts (Table 2).

### 2.3. Inhibitory effect of extracts from suosuo grape on the expression of HBsAg, HBeAg and HBV DNA replication

The synergistic effects of suosuo grape extract (A-VTT, B-VTP and C-VTF) inhibition of secretion of HBeAg by Hep G 2.2.15 cells could be examined using an experimental design. In the present study, all selected factors were examined using an orthogonal L _8_(2)^7^ test design. The total evaluation index was used to analysis by statistical methods. The results of the orthogonal test and extreme difference analysis are presented in Table 3. The analysis of variance was performed using the statistical software SPSS 13.0 and the results are listed in Table 4. The results showed that there had synergistic effect among VTT, VTP and VTF on inhibition of HBeAg secretion by Hep G 2.2.15 cells, and the strengths of inhibition were A×B×C > B×C > B > C, in turn. The optimal combination of extracts was A_2_B_1_C_1_: using VTT 20 μg/mL, VTF 50 μg/mL and VTP 50 μg/mL together on HepG2.2.15.

## 3. Experimental 

### 3.1. Plant material

Suosuo grapes were collected from Tulufan, Xinjiang Urghur autonomous region, in China, in May 2006. The plant materials were identified as the fruits of *Vitis vinifer* L by Yan Fu Zhang, Institute of Materia Medica of Xingjiang. A voucher specimen was deposited at the Institute of Materia Medica of Xinjiang in China.

### 3.2. Extraction and isolation

Five kilogram of the plant material was extracted with deionized water under reflux at 80 ºC for 6 h in several batches. The combined water extracts was evaporated under vacuum to specific gravity of 1.1-1.2, and ethanol about five times the original volume was added and kept at 4 for 24 h. After being filtered and being washed by ethanol, acetone, and diethyl ether, the precipitates was collected and redissolved in distilled water and treated with Sevag’s reagent several times to remove protein and dialyzed against deionised water for 24 h at 4 ºC. The polysaccharides (VTP) were obtained though further precipitated with ethanol. The gruffs was extracted with 95% ethanol under reflux at 80 ºC for 6 h in several batches. The extracts were combined, filtered and evaporated under vacuum. The concentrated extracts was diluted with water and successively treated with petroleum ether and EtOAc. Total triterpenes (VTT) was prepared by the method of 95% ethanol recrystallized from the EtOAc fraction. The mother liquor of VTT crystalline powder combined with supernatant of ethanol precipitation to be separated by AB-8 resin. After elution with deionized water and cleaning of impurities, the 50% ethanol eluent was collected to afford total flavonoids (VTF).

### 3.3. Oleanolic acid content of VTT

Chromatographic separation was achieved by using HPLC system consisting of a Shimadzu LC-10AD and YMC-Pack ODS-A column (150 × 4.6 mm, 5μm, with pre-column), the mobile phase consisted of methanol and H_2_O (86:14). Detection wavelength was 215 nm. The flow rate was 1.0 mL/min. Oleanolic acid content of VTT was 65.52 mg/100 mg.

### 3.4. Flavonoid content of VTF

Total flavonoid content in the extracts was determined using the method described in the Chinese Pharmacopeia [10]. Quantitation was based on the standard curve of rutin (0.2–1.0 mg/mL), dissolved in 95% ethanol. Flavonoids content was calculated with rutin as the standard and total flavonoid content of VTF was 35.28 mg/100 mg 

### 3.5. Experiment design

The group design method was used to observe the inhibitory effects of VTT (6.25, 12.5, 25, 50 μg/mL), VTF (12.5, 25, 50, 100 μg/mL), VTP (6.25, 12.5, 25, 50 μg/mL) and 3-TC (1, 10, 100 μg/mL) on HepG2.2.15 cells secreting HBsAg, HBVeAg and HBV DNA. The synergistic effect of three extracts from Suosuo grape on HBV was studied by orthogonal experiments designed as followed: three factors were determined according to screening samples (A-VTT, B-VTP and C-VTF); every factor was divided with two levels (Table 5). Interactions of the three factors and their main effects were examined, and errors obtained though re-experiments.

### 3.6. Cell culture

The HepG2.2.15 cell line which is stably transfected with HBV DNA, was grown in Dulbecco’s modified Eagle’ medium nutrient mixture containing geneticin (G418) 100 μg mL^-1^ with 10% (v/v) heat-inactivited fetal bovine serum (FBS), 1 × 10^5^ U mL^-1^ penicillin, 1.0 mg mL^-1^ streptomycin. Cells were incubated at 37 ºC in a humidified 5% CO_2_ atmosphere.

### 3.7. Cytotoxicity assays

The cytotoxic effects of VTT, VTP, VTF and 3-TC were evaluated by MTT essay [11]. Cells were seeded in 96-well culture plates at a density of 1 × 10^6^ cell mL^-1^ and cultured at 37 ºC for 48 h. Then the culture medium was removed and replaced with fresh medium supplemented with various concentrations of VTT, VTP, VTF and 3-TC every other day. After eight days of culture, the medium was discarded and 10 μL of a solution of 5 mg/mL 3-(4,5-dimethylthiazol-2-yl)-2,5-diphenyl-tetrazolium bromide (MTT) was added and the cultures were incubated for an additional 4 h. The supernatant was then removed and the cells were dissolved in DMSO (150 μL/well). The relative cell viability was quantitatively measured by scanning at 490 nm on an enzyme-linked immunosorbent assay (ELISA) reader (Bio-Tek ELX800; Bio-Tek Instruments Inc., Winooski, VT, USA) and the 50% inhibitory concentration (TC_50_) on cells was calculated by MTT assay as follows: cell inhibition (%) = (A_cell control_ – A_sample_) / (A_cell control_ – A_control_) ×100%.

### 3.8. HBsAg and HBeAg analysis

The HepG2.2.15 cells were plated at a density of 1 × 10^6^ cell mL^-1^ on 96-well cell culture plates and routinely cultured. Different concentrations of test samples were supplemented to the medium in triplicate after cells were plated at 37 ºC for 48 h. After incubation with test smples for eight days, the supernatants were collected and clarified by centrifugation at 5000×g for 5 min, HBsAg and HBeAg were determined by ELISA kits, performed according to the protocol provided with the kit (Ke Hua Biological Corp, Shanghai, China). The results were read at 450 nm by an ELISA reader (Bio-Tek ELX800; Bio-Tek Instruments Inc., Winooski, VT, USA). The inhibition of samples on HBsAg and HBeAg were calculated by formula as follows:

HBsAg (or HBeAg) inhibition (%) = (A_cell control_ – A_sample_) / (A_cell control_ – A_control_) × 100%, and the 50% inhibitory concentration (IC_50_) on cells was calculated. 

The therapeutic index (TI) was calculated by IC_50_ and TC_50_ (TI= TC_50_ / IC_50_) to judge the effect of test samples. TI > 2 as effective and low-toxicity; 1 < TI < 2 as low-effective and toxicity and TI < 1 expressed that sample have bigger toxicity and cannot be used as anti-HBV agent.

### 3.9. Detection of HBV DNA

The quantity of HBV DNA in the supernatant was detected by real-time PCR (ABI PRISM 7300 Sequence Detector, PE Biosystems) based on the TaqMan technology. Viral DNA was extracted from the culture supernatant and the amount of hepatitis B viral DNA was quantified using a diagnostic kit (Shenyou bio technology Co. Ltd., Shanghai, China). A series dilution of known amounts of HBV DNA was used as a control. The inhibitor rate of sample on HBV DNA was calculated by formula as following: the inhibitory rate (%) = (Copies_cell control_ –Copies_sample_) /Copies_cell control_×100%.

### 3.10. Statistical analysis

Data were expressed as mean±SD and were carried out using SPSS Version 13.0 software. Analysis of variance was performed by ANOVA procedures and *P* < 0.05 was considered to be statistically significant.

## 4. Conclusions 

The VTT, VTF and VTP from suosuo grape had antiviral effect *in vitro*. These active extracts could play an antiviral effect alone or by synergistic use. Further *in vivo* investigation to clarify their anti-HBV activities and the mechanisms of these bioactivities is required. 

## Figures and Tables

**Table 1:** Therapeutic index.

Sample	TC_50_(μg/mL)	HBsAg		HBeAg	
		IC_50_(μg/mL)	TI	IC_50_(μg/mL)	TI
VTT	160.61	62.54	2.57	89.28	1.80
VTP	1412.85	112.69	12.54	204.46	6.91
VTF	284.91	327.56	0.87	215.34	1.32
3-TC	244.77	315.37	0.78	9051.01	0.03

**Table 2:** The effect of extracts from *Vitis vinifer* L on HBV DNA replication

Sample	Concentration(μg/mL)	HBV DNA(copies/mL)	HBV DNA inhibition ratio (%)
VTT	50	(4.842 ± 4.457) × 10^5^	59.17
	25	(8.269 ± 2.002) × 10^5^	30.28
	12.5	(0.944 ± 1.460) × 10^6^	20.40
	6.25	(1.015 ± 5.230) × 10^6^	14.42
VTP	100	(4.823 ± 3.394) × 10^5^	59.33
	50	(7.525 ± 5.883) × 10^5^	36.55
	25	(0.873 ± 5.335) × 10^6^	26.39
	12.5	(1.158 ± 2.027) × 10^6^	2.36
VTF	100	(6.211 ± 1.910) × 10^5^	47.63
	50	(0.970 ± 0.432) × 10^6^	18.21
	25	(0.892 ± 2.872) × 10^6^	24.79
	12.5	(1.162 ± 0.201) × 10^6^	2.02
3-TC	100	< 10^3^	> 99.99
	10	< 10^3^	> 99.99
	1	< 10^3^	> 99.99
Cell group		(1.186 ± 0.226)×10^6^	

**Table 3:** Analysis of L_8_ (2)^7^ test results.

NO	A	B	A×B	C	A×C	B×C	A×B×C	HBeAg inhibition ratio(%)					
	1	2	3	4	5	6	7	X_1_	X_2_		X_3_		ΣX
1	1(50)	1(50)	1	1(50)	1	1	1	65.55	60.24		45.88		171.67
2	1(50)	1(50)	1	2(25)	2	2	2	46.29	49.39		54.45		150.13
3	1(50)	2(25)	2	1(50)	1	2	2	51.18	46.37		46.86		144.41
4	1(50)	2(25)	2	2(25)	2	1	1	38.53	29.96		41.22		109.71
5	2(25)	1(50)	2	1(50)	2	1	2	59.84	64.33		69.06		193.23
6	2(25)	1(50)	2	2(25)	1	2	1	48.08	37.31		35.59		120.98
7	2(25)	2(25)	1	1(50)	2	2	1	46.12	48.08		29.80		124.00
8	2(25)	2(25)	1	2(25)	1	1	2	64.08	58.94		47.58		170.60
Ⅰ	575.92	636.01	616.40	633.31	607.66	645.21	526.36						
Ⅱ	608.81	548.72	568.33	551.42	577.07	539.52	658.37						
R	32.89	87.29	48.07	81.89	30.59	105.69	132.01						

A, VTT; B, VTP; C, VTF.

**Table 4:** The analysis of variance of test results.

The source of variation	SS	υ	MS	F	Sig.
Total variation	2771.173	23			
Total inter-treatment	1968.782	7			
A	45.073	1	45.073	0.899	0.357
B	317.481	1	317.481	6.331	0.023
C	279.416	1	279.416	5.572	0.031
Frist interaction A×B	96.280	1	96.280	1.920	0.185
A×C	38.990	1	38.990	0.777	0.391
B×C	465.432	1	465.432	9.281	0.008
Second interaction A×B×C	726.110	1	726.110	14.479	0.002
Error	802.392	16	50.149		

A, VTT; B, VTP; C, VTF.

**Table 5:** Factors and levels for orthogonal test.

Variable	Level	
	1	2
A, VTT	50	25
B, VTP	50	25
C, VTF	50	25
